# p53 dynamics orchestrates with binding affinity to target genes for cell fate decision

**DOI:** 10.1038/cddis.2017.492

**Published:** 2017-10-19

**Authors:** Mengqiu Wu, Hui Ye, Zhiyuan Tang, Chang Shao, Gaoyuan Lu, Baoqiang Chen, Yuyu Yang, Guangji Wang, Haiping Hao

**Affiliations:** 1 Key Laboratory of Drug Metabolism and Pharmacokinetics, State Key Laboratory of Natural Medicines, China Pharmaceutical University, Tongjiaxiang #24, Nanjing, Jiangsu 210009, China; 2 Department of Nephrology, Children’s Hospital of Nanjing Medical University, Nanjing, Jiangsu 210008, China; 3 Department of Respiratory Medicine, Affiliated Hospital of Nantong University, Nantong, Jiangsu 226001, China

## Abstract

Emerging evidence support that temporal dynamics is pivotal for signaling molecules in orchestrating smart responses to diverse stimuli. p53 is such a signaling molecule that employs temporal dynamics for the selective activation of downstream target genes and ultimately for cell fate decision. Yet how this fine-tuned p53 machinery is quantitatively decoded remains largely unclear. Here we report a quantitative mechanism defining how p53 dynamics orchestrates with binding affinity to target genes for cell fate decision. Treating cells with a genotoxic drug doxorubicin at various doses and durations, we found that a mild and prolonged challenge triggered sequential p53 pulses and ultimately resulted in a terminal pulse enacting apoptosis in a comparable rate with that induced by an acute and high-dose treatment. To transactivate proapoptotic genes and thereafter executing apoptosis, p53 must exceed a certain threshold and accumulate for sufficient time at levels above it. Effective cumulative levels above the threshold, defined as E∫p53, but not the total accumulation levels of p53, precisely discriminate survival and apoptotic cells. p53 accumulation below this threshold, even with prolonging time to reach a total level comparable to that from the accumulation over the threshold, could not transactivate proapoptotic genes to which the binding affinity of p53 is lower than that of proarrest genes, and this property is independent of dynamic features. Our findings indicate that the dynamic feature *per se* does not directly control cell fate, but rather it orchestrates with the binding affinity to target genes to confer an appropriate time window for cell fate choice. Our study provides a quantitative mechanism unifying p53 dynamics and binding affinity to target genes, providing novel insights to understand how p53 can respond quantitatively to chemotherapeutic drugs, and guiding the design of metronomic regimens for chemotherapeutic drugs.

Cells use an efficiently and precisely controlled signaling network to sense and respond to endogenous and exogenous stresses.^1^ In response to stress, signaling molecules can be regulated at transcriptional, translational, and posttranslational levels^2, 3^ and modulated by the change of protein–protein interactions,^4^ spatial location,^5, 6^ and three-dimensional structure^7, 8^ to orchestrate fine-tuned responses to different types and extents of stresses and thereby ensuring appropriate functional adaptations. In addition to all these ‘static’ mechanisms, emerging evidence indicates that signaling molecules may decode their capacity of selective responses to diverse stimuli via dynamic features.^9^ Representative signaling molecules such as p53,^10, 11, 12, 13, 14^ NF-*κ*B,^6, 15^ ERK,^16, 17^ and MSN2^5, 18^ can be manifested as pulses, monotonic pulse and sustained activation for smart functional choice. The encoding mechanisms of the dynamic features of signaling molecules, which explain how differential dynamics are produced from different stresses, have been clarified via exploring the feedback regulating loops of signaling molecules.^14, 19^ In contrast, the decoding mechanisms underlying how differential dynamics of signaling molecules are translated to different functional outcomes remain largely unexplored.

The tumor-suppressor p53 is a central player in sensing various types and extents of stresses leading to differential cell fates ranging from transient responses such as cell cycle arrest and DNA repair to terminal events, including senescence and apoptosis.^20, 21, 22^ p53 can be activated in either pulsed or sustained mode,^10, 23^ and it is believed that pulsed p53 leads to transient response such as cell cycle arrest and DNA repair, while sustained activation of p53 results in irreversible cell fates of senescence and apoptosis.^10, 23^ Subsequently, the dynamic features of p53 have been intensively explored for the explanation of the cell-to-cell variation in response to chemotherapeutic drugs^24^ and for guiding the identification of a schedule-dependent combinatory therapeutic approach.^25^ However, the exact mechanism underlying how p53 quantitatively senses stimuli for cell fate decision remains largely unclear. Here we report a mechanism that unifies p53 dynamics and binding affinity to target genes conferring appropriate time windows for cell fate choice. We found that to transactivate proapoptotic genes and thereafter enacting cell apoptosis, p53 must exceed a certain threshold and accumulate for sufficient time at levels above this threshold. Effective cumulative levels above this threshold, defined as E∫p53, but not the total accumulation levels of p53, precisely discriminate surviving and apoptotic cells. In contrast to previous concept that pulsed and sustained activation of p53 leads to differential cell fates, we validated that pulsed activation of p53 with prolonging time ultimately resulted in a terminal pulse rendering a fast accumulation to break through the threshold to executing apoptosis. Our findings indicate that the dynamic feature *per se* does not directly control cell fate, but rather it orchestrates with the binding affinity to target genes to confer an appropriate time window for cell fate choice.

## Results

### Distinct p53 dynamics lead to comparable cell apoptosis

**Figure 1:**
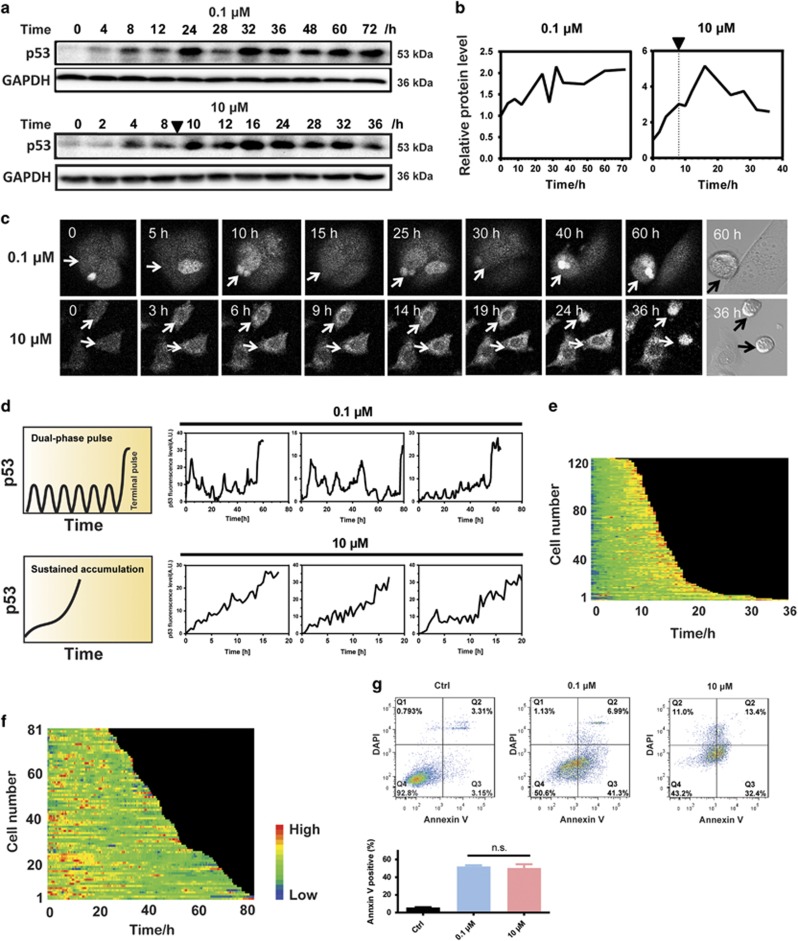
Prolonged pulsatile and sustained activation lead to comparable cell apoptosis. (**a**) Immunoblots of p53 dynamics induced by a weak and prolonged stimulus (0.1 *μ*M Dox treatment for 72 h) and a strong and acute stimulus (10 *μ*M Dox treatment for 8 h). Triangle indicates the time when Dox was withdrawn. (**b**) Quantification of p53 abundance. Triangle and dashed line indicate the time when Dox was withdrawn. (**c**) Time-lapse images of representative MCF7 cells (indicated by arrows) expressing p53-Venus following Dox treatment. Cell apoptosis was identified visually by bright-field channel, and the corresponding p53-Venus levels were quantified from the fluorescence intensity. (**d**) Representative single-cell traces of MCF7-p53-Venus cells following Dox treatment. Corresponding p53 dynamic patterns are summarized on the left. (**e** and **f**) p53-Venus fluorescence for each cell cells under 10 *μ*M (**e**) or 0.1 *μ*M (**f**) Dox treatment were measured by time-lapse microscopy, floor-subtracted and drew into heat maps. p53 traces of individual cells are arranged from top to bottom by the lifespan. (**g**) Weak and prolonged stimulus (0.1 *μ*M Dox treatment for 72 h), and a strong and acute stimulus (10 *μ*M Dox treatment for 8 h and cultured to 48 h) lead to comparable apoptotic rate as shown by the flow cytometry analysis using Annexin V/DAPI staining. Control cells were cultured in drug-free medium for 4 days. NS, not significant

To elucidate the exact mechanism of how p53 dynamics controls cell fate, the responses of p53 to different doses of a genotoxic drug doxorubicin (Dox) and their association with the cell fates were determined. In the cell population study, the low-dose treatment of Dox triggered a pulsatile behavior of p53 protein levels, whereas the high dose induced a sustained activation of p53 (Figures 1a and b), similar to that observed from *γ* and UV irradiation, respectively.^11^ Because cell population-based observation may mask p53 dynamical patterns in single cells,^9^ we quantified the p53 protein dynamics at single-cell level by measuring Venus fluorescence in the nucleus using clonal MCF7 cells expressing p53-Venus via time-lapse microscopy (Supplementary Movies S1–S3). The p53-Venus reporter construct mimicked the dynamic behaviors of the endogenous p53 protein.^13^ The time-lapse recording of p53 protein in individual cells confirmed that the prolonged low-dose treatment of Dox induced a series of pulses, and acute treatment with high dose led to a sustained induction of p53 (Figures 1c–f,Supplementary Movies S1–S3). Intriguingly, the long duration recording of single cells enabled us to discover a dual-phase pattern of p53 pulses. In response to prolonged low-dose treatment of Dox, p53 in individual cells first initiated a series of pulses with fixed amplitude and then abruptly increased to a high-amplitude level enacting apoptosis (Figures 1c and d and Supplementary Movies S1). We defined the abrupt increase of p53 levels after a series of pulses as ‘terminal pulse’ (Figure 1d). Similar pattern was found in response to etoposide treatment (Supplementary Figure S1), suggesting that the dual-phase p53 pulse is not limited to Dox treatment. In contrast to previous concept that pulsed and sustained activation of p53 leads to differential cell fates,^10, 26^ we found that, with the prolonged treatment of Dox at a dose >0.05 *μ*M, dramatic cell cycle arrest was observed on the first day and apparent apoptosis appeared on the fourth day after the treatment (Supplementary Figure S2A). And the prolonged treatment with Dox at 0.1 *μ*M resulted in comparable apoptosis of cells than that treated with Dox at 10 *μ*M for 8 h as measured by flow cytometry^27^ (Figure 1g,Supplementary Figures S2A and B), in spite of their distinct features of p53 dynamics (Figures 1c and d), which are clearly shown by the heat maps recording p53-Venus abundance over time in individual cells (Figures 1e and f). The apoptotic fate induced by the two treatments was further validated by the increased levels of PUMA and BAX (Supplementary Figure S2C). These results combinatorially indicate that the dynamic feature of p53 *per se* might not directly control cell fates.

### Effective cumulative levels of p53 above a threshold control cell fate

**Figure 2:**
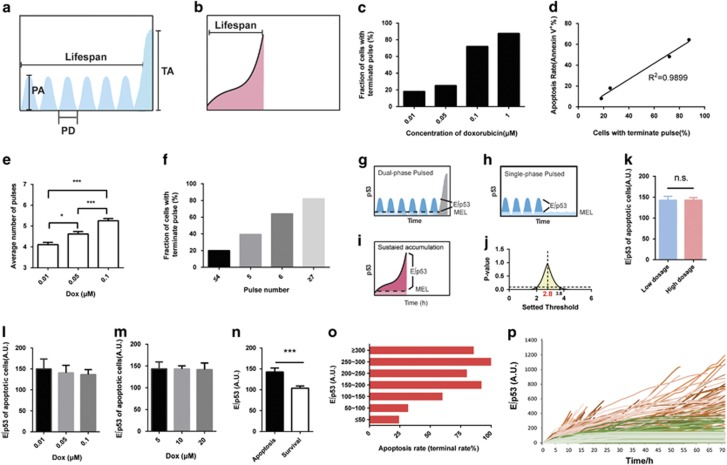
Integral cumulative levels of p53 over a threshold control cell fates. (**a**) Prolonged low-dose treatment of Dox (0.1 *μ*M) initiated a series of pulses and then abruptly increased to a high-amplitude level of p53 (defined as ‘terminal pulse’). Metrics used to describe the dynamic pattern are pulse amplitude (PA), lifespan, pulse duration (PD), and terminal amplitude (TA). (**b**) Acute Dox treatment (10 *μ*M) induced a sustained accumulation of p53. (**c**) The fraction of cells showing terminal pulses within 96 h under Dox treatments as specified. Number of cells (*n*)=121 (0.01 *μ*M), *n*=127 (0.05 *μ*M), *n*=118 (0.1 *μ*M) and *n*=54 (1 *μ*M). (**d**) Correlation analysis of apoptosis rate (Annexin V-positive rate) and the fraction of cells showing terminal pulse within 96 h (*n*=420 in total for single-cell analysis). (**e**) The average number of pulses in response to different dose of Dox stimuli. Number of cells (*n*)=121 (0.01 *μ*M), *n*=127 (0.05 *μ*M), and *n*=118 (0.1 *μ*M). Data are represented as mean±S.E.M.; *P<0.05; ***P<0.001. (**f**) The fraction of cells showing terminal pulse after corresponding number of p53 pulses. The cell fraction in each bin was calculated by summarizing the number of cells showing corresponding number of pulses followed by dividing them by the total number of cells included in panel (**e**) (*n*=366). (**g**–**j**) Schematic illustration of a predicted threshold in initiating apoptosis. The presumed threshold is defined as MEL and the p53 accumulation above MEL as effective cumulative level of p53 (E∫p53) (**g**–**i**). The threshold was searched at a step of 0.1 A.U. from 0 to 5 A.U., and the value that delivered identical E∫p53 with the maximum *P*-value was designated as the threshold (**j**). (**k**) Average levels of E∫p53 from apoptotic cells treated with low (0.01–0.1 *μ*M for 72 h, *n*=139) or high dose (5–20 *μ*M for 8 h and cultured to 48 h, *n*=214) of Dox (threshold=2.8 A.U.). Data are represented as mean±S.E.M. (**l** and **m**) Average levels of E∫p53 from apoptotic cells treated with low (*n*=139) and high concentration (*n*=214) of Dox are shown, respectively. (**n**) E∫p53 level in apoptotic (*n*=227) and survival cells (*n*=139) within 96 h. Error bars represent S.E.M.; ***P<0.001. (**o**) Apoptotic rate (%) of cells that display corresponding levels of E∫p53. (**p**) Single-cell traces of E∫p53 in surviving cells (green) and apoptotic cells (red). Single-cell data for panels (**o**) and (**p**) were calculated using cells treated with 0.01, 0.1 and 10 *μ*M Dox (*n*=367 in total). Cell fate can be identified visually by morphological changes observed using the bright-field channel. NS, not significant

To fully characterize the two-phase p53 dynamics, several parameters were designed (Figures 2a and b). Of interest, the average pulse amplitude, lifespan, and terminal amplitude held constant across the individual cells treated with differential doses of Dox (Supplementary Figures S3A–C), suggesting an excitable mechanism for triggering terminal pulse, similar to that observed for p53 pulses induced by radiomimetic drug neocarzinostatin and *γ* irradiation.^11^ In contrast, the appearance of p53 terminal pulse increased in a dose-dependent manner (Figure 2c) and is linearly correlated with the apoptotic rates determined by flow cytometric analysis using Annexin-V/DAPI staining (Figure 2d), supporting that terminal pulse is a direct indicator of cells enacting apoptosis. In line with that observed from *γ* irradiation stimuli,^14, 28^ the average number of pulses increased with the increasing dose of Dox (Figure 2e). Intriguingly, the cell fractions with terminal pulse increased with the number of pulses and >60% of cells with 6 pulses resulted in a terminal pulse, although the number of pulses for eliciting the terminal pulse varied in individual cells (Figure 2f). We next compared various parameters of p53 dynamics between apoptotic and survival cells by pooling all sets of treatments together. Survival cells showed single-phase pulsatile behaviors of p53 without terminal pulse seen in apoptotic cells (Figures 1d and 2g, Supplementary Movies S1 and S2). No difference was observed for the amplitude and duration of p53 pulses and also the integral accumulation levels (∫p53) between apoptotic and survival cells (Supplementary Figures S3D–F). In contrast, the number of pulses in apoptotic cells was much higher than that in survival cells (Supplementary Figure S3G). As a support, the cell fractions with higher number of p53 pulses increased with the dose of Dox (Supplementary Figures S3H–J), supporting that the number of pulses is sensitive to the extent of Dox-induced DNA damage. These results are in accordance with a previous theoretical prediction that the number of pulses might be an indicator of cell fate decision.^29^

We then asked why the increased number of p53 pulses is closely associated with the appearance of terminal pulse and why differential features of p53 dynamics, pulsed and sustained activation, resulted in a comparable rate of cell apoptosis. The integral accumulation of p53 (∫p53) is almost identical between survival and apoptotic cells (Supplementary Figure S3F).The value of ∫p53 is even higher in the cells with low-dose Dox treatment than that with high-dose treatment (Supplementary Figure S3K). This is probably due to the much longer lifespan of p53 in cells under low-dose treatment before they enact apoptosis compared with the apoptotic cells stimulated by an acute, high-dose treatment. Consequently, the absolute integral level of p53 accumulation is apparently not a determining factor of cell fate decision. In view that p53 at basal level and the spontaneous pulse in unstressed conditions dose not transactivate its downstream target genes,^12^ we reasoned that p53 accumulation below a certain threshold might not contribute to the activation of proapoptotic genes (Figure 2g). This concept is similar to the well-defined pharmacological concept that a drug should exceed its minimal effective concentration to elicit an observable efficacy. Because the dual-phase pulsatile and the sustained activation of p53 resulted in a comparable rate of cell apoptosis (Figure 1g and Supplementary Figure S2), we hypothesized that the integral accumulation levels of p53 above a certain threshold could also be comparable between these two types of dynamics. We defined this threshold as ‘minimal effective level’ (MEL) and the p53 accumulation above the threshold as ‘effective cumulative p53’ (E∫p53) (Figures 2g–i). We then developed a mathematical model to determine the threshold by assuming an identical average E∫p53 of individual apoptotic cells between these two treatments. Ranging from 0 to 5 A.U., the threshold was proposed at a step of 0.1 A.U., and the corresponding E∫p53 values were calculated. Results showed that no significant difference was observed for the E∫p53 of apoptotic cells between the two groups from 2 to 3.8 A.U. (Figure 2j). The maximum *P*-value appeared when the threshold was set at 2.8 A.U. (Figures 2j and k). The E∫p53 of apoptotic cells was almost identical among the groups of cells with low-dose treatments, which produced a number of p53 pulses and terminal pulse (Figure 2l); similar result was observed for the set of high-dose treatments characterized with sustained p53 activation (Figure 2m). In contrast, the E∫p53 in apoptotic cells was significantly higher than that in survival cells (Figure 2n). By calculating the E∫p53 of the apoptotic cells from all groups of treatments, we found that, although the E∫p53 level varied dramatically among individual cells, higher E∫p53 value is closely associated with higher apoptotic rate (Figure 2o). Moreover, time-dependent E∫p53 curves clearly differentiate the apoptotic cells from the survival cells with an accuracy of ~76% (Figure 2p). Because some other p53-independent factors may trigger cell apoptosis, the overlap of E∫p53–time curves between apoptotic and survival cells is acceptable.^30, 31^ All these results support our hypothesis that, regardless of dynamic patterns, p53 should exceed an MEL and maintain for sufficient time to reach certain levels of E∫p53 and thereafter enacting apoptosis.

### MDM2 repression promotes cell apoptosis without the change of E∫p53

**Figure 3:**
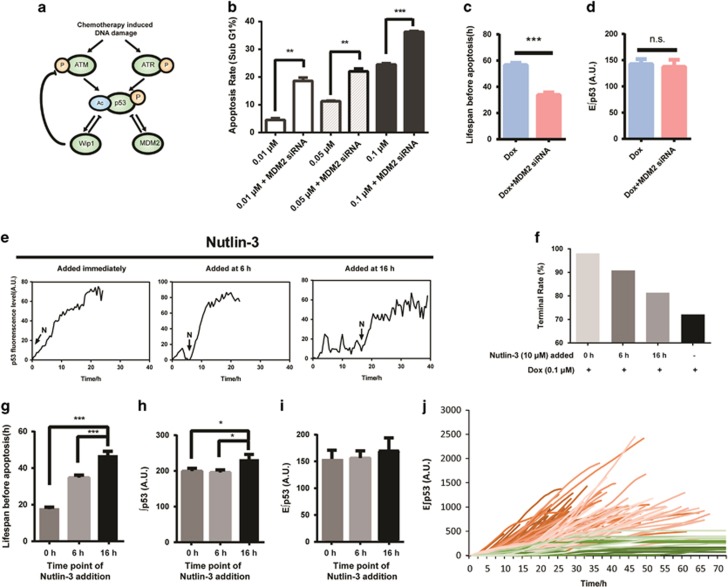
MDM2 repression facilitates cell apoptosis without the change of E∫p53. (**a**) Regulatory network of p53 in response to chemotherapy. (**b**) Apoptotic rate of cells treated with Dox with or without MDM2 silencing for 72 h. Data are represented as mean±S.E.M.; **P<0.01; ***P<0.001. (**c** and **d**) Lifespan and E∫p53 level of cells treated with 0.1 *μ*M Dox combined with or without MDM2 silencing. Data are represented as mean±S.E.M.; ***P<0.001. (**e**) Representative single-cell traces of MCF7-p53-Venus cells treated with 0.1 *μ*M Dox combined with Nutlin-3 (10 *μ*M) added immediately or at 6 and 16 h, following Dox treatment, respectively. ‘N’ with an arrow in the figure indicates the time when Nutlin-3 (N) was added. (**f**) The rate of cells exhibiting terminal pulse (terminal rate) under different treatments within 96 h (*n*=293 in total). (**g**–**i**) Lifespan, ∫p53 and E∫p53 levels of apoptotic cells treated with 0.1 *μ*M Dox and co-administered with Nutlin-3 added to medium at the indicated time points (*n*=175 in total). Error bars represent S.E.M.; *P<0.05; ***P<0.001. (**j**) Single cell traces of E∫p53 in response to 0.1 *μ*M Dox treatment co-administered with Nutlin-3. Green, surviving cells (*n*=17); red, apoptotic cells (*n*=175). NS, not significant

Subsequently, we sought to determine whether the concept of E∫p53 still holds useful when the dynamic features of p53 were artificially changed. For this purpose, we first determined the encoding mechanism of Dox-triggered dual-phase p53 dynamics (Figure 3a). The protein levels of p53 encoding network were determined in MCF7 cells treated with increasing concentrations of Dox at 72 h, the time when terminal pulses appeared in a large proportion of cells. As indicated by phosphorylated *γ*H_2_AX, a sensitive marker of the DNA double-strand breaks, the degree of DNA damage increased with increasing dose of Dox (Supplementary Figure S4A). The phosphorylation of both ataxia telangiectasia and Rad3-related kinase (ATR) and ataxia telangiectasia mutase kinase (ATM) were increased dose-dependently. Phosphorylated ATM and ATR not only increased p53 expression but also the p53 acetylation level (Supplementary Figure S4A), which is important for the nuclear translocation of p53 (Supplementary Figure S4B) and subsequent activation of the proapoptotic genes transcriptionally.^32, 33^ Concomitantly, the negative regulator, murine double minute 2 (MDM2), decreased at the 0.1 *μ*M dosage of Dox, while the other negative regulator, wild-type p53-induced phosphatase gene 1 (Wip1), showed almost negligible change at the same time point (Supplementary Figure S4A). Therefore, the upregulation of upstream activator and downregulation of negative regulator of p53 explained the presence of terminal pulse in the dual-phase p53 dynamics as we observed.

Of particular interest, the ratio of p-ATM to MDM2 increased with the time of Dox treatment (Supplementary Figures S4C–E). It seems that if the DNA damage could not be repaired after sequential p53 pulses, cells might ultimately break a certain barrier (the balance between upstream kinases and negative regulators), rendering an abrupt accumulation of p53 to enact apoptosis. Thereafter, we chose to target MDM2 to mimic the disruption of p53 balance for further validation of the concept of E∫p53. The siRNA of MDM2 and the typical inhibitor Nutlin-3 that binds to MDM2 and stabilizes p53 were employed. Specifically, Nutlin-3 was added at 0, 6, or 16 h following the 0.1 *μ*M Dox treatment to induce differential p53 dynamic features. Silencing of MDM2 following Dox treatment promoted the accumulation of p53 and triggered an early onset of p53 terminal pulse (Supplementary Figures S4F and G), accompanying with a significantly increased apoptotic rate (Figure 3b) and shortened cell lifespan before apoptosis (Figure 3c), while the E∫p53 of apoptotic cells held constant (Figure 3d). In addition, we found that the Nutlin-3 treatment immediately stimulated the accumulation of p53 (Figure 3e). The earlier addition of Nutlin-3 led to a relatively higher rate of cell apoptosis (Figure 3f) and shortened cell lifespan (Figure 3g). Notably, although the total accumulating levels of p53 varied in apoptotic cells induced by different treatments (Figure 3h), the E∫p53 held constant across all treatments (Figure 3i). More importantly, the time-dependent E∫p53 curves efficiently discriminated the apoptotic and survival cells (Figure 3j). Additionally, the silencing of another p53-negative regulator, Wip1, achieved similar results as that observed for MDM2-interfered cells, characterized with increased rate of p53 accumulation and cell apoptosis (Supplementary Figures S4H–J). These results support that, regardless of dynamic features, E∫p53 is a useful and reliable indicator for cell fate decision.

### E∫p53 underlies the transactivation of proapoptotic genes

**Figure 4:**
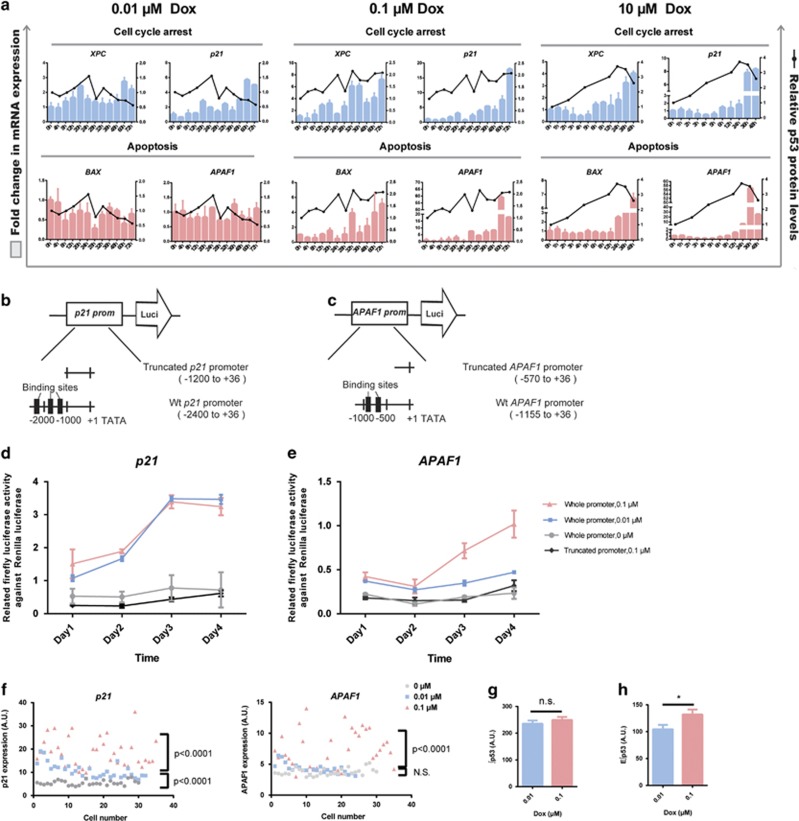
E∫p53 differentiates the transactivation of different sets of target genes. (**a**) Expression of p53 target genes. mRNA levels of target genes under the indicated conditions are shown in histograms. Genes are grouped into cell cycle arrest- and apoptosis-related genes. Data of transcripts are mean±S.D. Relative p53 protein levels normalized to GAPDH are shown as black lines. (**b**) Luciferase reporter gene assay, using wild-type *p21* promoter (−2400 to +36 bases, pGL3-*p21*-Luci) and a truncated *p21* promoter (−1200 to +36 bases) with deletion of three reported p53-binding sites. (**c**) Luciferase reporter gene assay, using wild-type *APAF1* promoter (−1155 to +36 bases, pGL3-*APAF1*-Luci) and a truncated *APAF* promoter (−570 to +36 bases) with deletion of two reported p53-binding sites. (**d** and **e**) Relative luciferase activity in MCF7 cells transfected with *p21* (**d**) and *APAF1* (**e**) promoter reporters. Dual luciferase reporter assay was performed by transfecting *p21* or *APAF1* luciferase reporter construct together with pRL-SV40 vector as an internal standard. Truncated luciferase reporter construct was transfected as negative control. Cells were treated with 0.01 and 0.1 *μ*M Dox for 1–4 days. Data are presented as mean±S.E.M. from three independent experiments. (**f**) FISH analysis of *APAF1* and *p21* transcripts after 0.01 and 0.1 *μ*M Dox treatment for 72 h. *n*=30 (0 *μ*M), *n*=31 (0.01 *μ*M), and *n*=35 (0.1 *μ*M). Unpaired Student’s *t*-test was conducted to determine the significance. (**g** and **h**) Average cumulative level of p53 (∫p53) (**g**) and effective cumulative level of p53 (E∫p53) (**h**) levels in cells treated with 0.01 *μ*M (*n*=121) and 0.1 *μ*M (*n*=118) Dox within 96 h. Data are represented as mean±S.E.M.; *P<0.05; NS, not significant

We next explored how E∫p53 controls the transactivation of p53 target genes and why distinct p53 dynamics led to the same apoptosis rate. In response to 0.01 *μ*M Dox treatment that delivered p53 pulses and led to cell cycle arrest, the cell cycle arrest genes (*XPC* and *p21*) showed an oscillatory induction that resembled pulsatile p53 dynamics. In contrast, the target genes encoding apoptotic proteins (*BAX* and *APAF1*) were not induced by pulsatile p53 dynamics (Figure 4a, left panel). On the contrary, the transcripts encoding apoptotic proteins in the cells treated with 0.1 *μ*M Dox showed a delayed induction that mirrored the appearance of terminal pulse, and the expression patterns and levels of these proapoptotic genes were quite similar to that of the sustained activation of p53 induced by acute and high-dose Dox treatment (Figure 4a, middle and right panels). The terminal pulsed activation of p53 also led to upregulation of cell cycle arrest genes comparable to that of sustained activation of p53 to an extent much higher than that by single phase of p53 pulses induced by 0.01 *μ*M Dox treatment (Figure 4a). To further validate these findings, we employed a luciferase reporter assay of p53 binding to the promoter of proarrest *p21* and proapoptotic *APAF1*. We found that the upregulation of *APAF1* was observed by 0.1 *μ*M Dox but not by 0.01 *μ*M Dox treatment (Figures 4b–e). These results support that cells with sequential pulses followed by a terminal pulse are capable of transactivating the proapoptotic genes. We further tested this in individual cells by a fluorescence *in situ* hybridization (FISH) analysis of the transcripts of *p21* and *APAF1*. The FISH assay showed that both 0.01 and 0.1 *μ*M Dox treatment upregulated *p21* but only the treatment with 0.1 *μ*M Dox upregulated *APAF1*. Moreover, the upregulation of *p21* in cells with 0.1 *μ*M Dox was much higher than that by 0.01 *μ*M Dox treatment (Figure 4f). Although the total accumulation levels of p53 between these two sets of treatment were almost identical (Figure 4g), the E∫p53 was significantly higher in cells treated with 0.1 *μ*M Dox than those by 0.01 *μ*M Dox (Figure 4h). These results reiterate that p53 should exceed a threshold (MEL) and accumulate for sufficient time to transactivate the proapoptotic genes.

**Figure 5:**
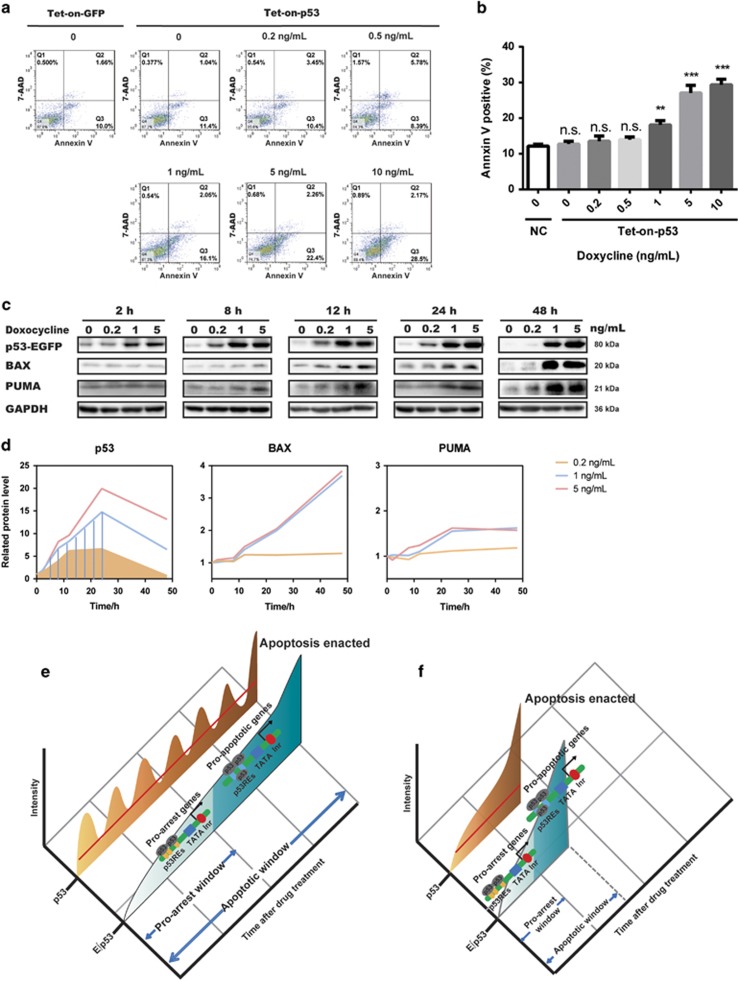
Doxycycline-inducible p53 expression system confirmed the established threshold of cell fate decision. (**a** and **b**) Flow cytometric analysis of apoptosis by Annexin V/7-AAD staining using tet-on-strict-p53-transfected MCF7 cells induced with 0–10 ng/ml doxycycline for 4 days. Tet-on-strict-GFP-transfected MCF7 cells were used as a control. Quantitation of the percentages of Annexin V-positive cells is shown in panel (b). Data are presented as mean±S.D. from three independent experiments. Unpaired Student’s *t*-test was conducted to determine the significance; *P<0.05, **P<0.01. (**c** and **d**) Western blotting analysis of p53 and apoptosis-associated proteins in MCF7 cells transfected with tet-on-strict-p53 plasmid and induced with 0.2–5 ng/ml doxycycline for the indicated duration. Quantification of p53, BAX, and PUMA protein abundance is shown in panel (**d**). Shaded areas indicate the cumulative level of p53 at corresponding time points. (**e** and **f**) Model illustrating how E∫p53 controls cell fate. Prolonged low-dose treatment of Dox initiated a series of pulses followed by a terminal pulse with increased amplitude (**e**). Acute and high-dose Dox treatment induced a sustained accumulation of p53 (**f**). E∫p53, the cumulative level of p53 above a MEL, discriminates the activation of different sets of target genes and thereby differentiates cell fate choice. NS, not significant

Because Dox-induced DNA damage might be complicated by other p53-independent factors, we further tested the threshold enacting apoptosis in an established doxycycline-inducible p53 expression system (Supplementary Figure S5A).The p53-inducible system had been previously validated to trigger different cell fates at different p53 expression levels, eliminating the influence of other variables.^34^ We treated the cells with doxycycline from 0 to 10 ng/ml to ascertain the threshold of p53 (Supplementary Figure S5B) and the corresponding cell fates (Figures 5a and b). Significant cell apoptosis was observed at a dose of doxycycline >1 ng/ml (Figures 5a and b). Three representative concentrations (0.2, 1, 5 ng/ml) of doxycycline were further tested for the time-dependent induction of p53 and its downstream proapoptotic proteins, BAX and PUMA (Figures 5c and d). Doxycycline at 0.2 ng/ml was able to induce the upregulation of p53 and sustain it for 24 h, whereas p53 at this level was not able to transactivate BAX and PUMA. With the increase of concentration, transfected p53 was induced to a relatively higher level and sustained for longer duration. Concentration of doxycycline >1 ng/ml was able to induce a stable upregulation of BAX and PUMA (Figures 5c and d), in agreement with the observed cell apoptosis (Figures 5a and b). Intriguingly, the ∫p53 within 48 h under 0.2 ng/ml treatment was comparable with that of 1 ng/ml treatment within 24 h (Figure 5d, shaded areas), reiterating ∫p53 cannot determine cell fates. Collectively, these results validate that a threshold exists for p53 to decide when and whether to activate the proapoptotic genes.

## Discussion

It is well established that p53 orchestrates a fine-tuned network for selective transactivation of its target genes and cell fate decision via multiple mechanisms.^30^ We reason that this well-tuned machinery must be operated in a quantitative manner. Here we have shown how p53 dynamics work quantitatively to differentiate the transactivation of different sets of target genes for cell fate decision. We provided a unified mechanism in understanding how p53 dynamics intertwines with its binding affinity to target genes in orchestrating an appropriate time window for cell fate decision (Figures 5e and f). It is not the absolute cumulative levels of p53 but rather the effective cumulative levels above a certain threshold that controls cell fates. Thus, in the conditions of mild stress, although the levels of p53 might periodically exceed the threshold, the accumulating time and the corresponding effective cumulative level is insufficient to transactivate proapoptotic genes. However, provided that the DNA damage persists and cells fail to repair the damaged DNA after sequential p53 pulses, the balance between the positive and negative regulating signals encoding p53 would be ultimately disrupted to elicit a terminal pulse enacting an irreversible cellular apoptosis. Therefore, the pulsatile feature in combination with the threshold mechanism may act together to orchestrate an appropriate time window for cells to recover from the fixable DNA damage (Figure 5e). This model may also explain why, in the sustained activation of p53, cells are more inclined to enact apoptosis; a faster rate of accumulation means an earlier breakthrough of the threshold and an earlier reach of the E∫p53 enacting apoptosis (Figure 5f).

The mechanism of p53 in controlling cell fates might be complicated by many other factors such as the posttranslational modification, protein–protein interactions, the accumulating threshold of its downstream target genes, and the interference of antiapoptotic signals. However, our results together with previous findings indicate that the rate, mode, time of accumulation, and integrally the E∫p53 might be important initiating factors in decoding the p53 complex network in cell fate choice.

Oscillatory dynamics is increasingly recognized as an important dimension explaining how signaling molecules can adaptively regulate diverse cell fates/functions.^9, 35, 36^ Moreover, the dynamic properties have been seen in an increasing list of protein signal molecules^37, 38^ and other molecules, such as RNA^39^ and calcium.^40^ The proposed model of p53 dynamics orchestrating with binding affinity to downstream target genes for precise control of cell signaling and fates may be also applicable to other transcriptional factors. We propose that the intensive and quantitative exploration of dynamic features is the key step for making these signaling molecules druggable. Furthermore, our findings that the prolonged treatment of very low dose of a chemotherapeutic drug, Dox, reached comparable apoptotic rate with that of high-dose treatment because of identical E∫p53 may have translational significance, supporting a low-dose and prolonged treatment regimen rather than the currently applied maximum tolerated dose (MTD) strategy, which usually leads to serious side effects and frequent drug resistance.^41, 42^ Clinically, the uninterrupted treatment with low doses of chemotherapeutic drug below the MTD with no drug-free break is referred to as metronomic chemotherapy.^43^ Such a treatment is attracting growing interests and holds promise to confer better clinical efficacies and less toxic effects of chemotherapeutic drugs as compared with that by conventional MTD regimen.^44, 45, 46^ Our findings provide a mechanistic support of the metronomic chemotherapy and, moreover, may ignite new idea of optimizing clinical regimen design for chemotherapeutic drugs.

## Materials and methods

### Time-lapse microscopy

For live-cell imaging, cells were grown on cell imaging glass-bottom plates (Eppendorf, Hamberg, German) in phenol red-free RPMI medium 1640 (Gibco, MA, USA) supplemented with 10% fetal calf serum, 100 U/ml penicillin, 100 *μ*g/ml streptomycin, and 250 ng/ml fungizone (HyClone, Logan, UT, USA). Cells were treated with Dox (Sigma, St. Louis, MO, USA) and Nutlin-3 (Selleck Chemicals, Houston, TX, USA) 24 h later and imaged with a customized Zeiss 700 confocal microscopy (Zeiss, Jena, Germany) on which the stage was surrounded by an enclosure to maintain constant temperature (37 °C), CO_2_ concentration (5%), and humidity. Images were acquired every 30 min for 2–4 days with a × 20 objective and a 488-nm diode laser. Apoptotic cells can be identified visually by bright-field channel, and the corresponding p53-Venus levels were quantified from the fluorescence intensity. Morphological changes observed during cell apoptosis include cell shrinkage, loss of focal adhesions, and the formation of cell membrane buds or blebs. We identified cellular apoptosis based on the presence of these key morpholigcal changes.^47^ The apoptotic rate or terminal rate were thus calculated by dividing the number of identified apoptotic cells or cells that accompanied with a terminal pulse by the total number of cells observed in microscopic experiments as previously described.^10, 11, 24, 26, 48^

### Flow cytometric analysis of cellular apoptosis

Cellular apoptotic rate was quantified by flow cytometry using Annexin V-Allophycocyanin (APC)/4',6-diamidino-2-phenylindole (DAPI) or Annexin V-APC/7-amino-actinomycin D (7-AAD) double staining. Data were acquired on a BD FACSVerse (Becton and Dickinson, Franklin Lakes, NJ, USA).

### Cumulative and effective cumulative p53 levels

Basal levels of p53-Venus fluorescence for each cell were measured before the treatment of Dox and subtracted from the entire time series. Cumulative p53 levels for each floor-subtracted trace were then calculated using trapezoidal integration with MatLab (Mathworks, Natick, MA, USA). Effective cumulative p53 levels were calculated by subtracting the area below the minimum effective level, that is, 2.8 A.U. as calculated by mathematical modeling in our case.

### Dual luciferase assay

The APAF1 luciferase reporter construct (pGL3-APAF1) was constructed by subcloning the APAF1 promoter (−1155 to +36) upstream of a firefly luciferase reporter using pGL3-Promoter vector (Promega, Madison, WI, USA) through Kpn I-Hind III sites. The truncated construct pGL3-APAF1 that miss two reported p53-binding sites includes sequence −570 to +36.^49^ Similarly, pGL3-p21 contains sequence −2400 to +36, whereas the corresponding truncated construct that miss three reported p53-binding sites contains −1200 to +36.^50^ MCF7 cells were transfected in 96-well plate with 180 ng firefly luciferase construct and 20 ng Renilla luciferase reporter plasmid pRL-SV40 vector, which was utilized as an internal standard, by Lipofectamine 2000 (Invitrogen, Carlsbad, CA, USA). The cells were then treated by 0.01 and 0.1 *μ*M Dox 24 h posttransfection and harvested for analysis using a Dual-Luciferase Assay Kit (Promega).

### RNA FISH microscopy

After the treatment of Dox, cells were fixed with 4% formaldehyde in DEPC water for 20 min followed by overnight incubation with 75% ethanol at 4 °C. FISH was performed as previously described.^51^ Resultant fluorescence images were captured using a Zeiss 700 confocal microscope with a × 63 oil objective (Zeiss, Jena, Germany). Gene expression levels of *p21* and *APAF1* were quantified using average cytoplasmic fluorescence at × 20 magnification. Probes for single-cell detection of *p21* and *APAF1* were synthesized by BersinBio, Guangzhou, China, and sequences are listed in Supplementary Table S1.

### pLVX-Tet-On-Tight-p53 construction and transfection

The pLVX-Tet-On-Tight-p53*EGFP-PGK-Puro and pLVX-Tet-On-Tight-EGFP-PGK-Puro (control) plasmids were generated following standard cloning procedures by Biowit Technologies, Shenzhen, China. The p53*EGFP coding sequence was cloned from an established construct of pEGFP-N1-TP53. MCF7 cells were seeded and cultured on six-well plates (50 000 cells/well) for 24 h and transfected with Tet-on-Tight-p53 or control plasmids using Lipofectamine 3000 (Invitrogen) according to the manufacturer’s instructions.

## Publisher’s Note

Springer Nature remains neutral with regard to jurisdictional claims in published maps and institutional affiliations.
