# Supplementary material for: p53 dynamics orchestrates with binding affinity to target genes for cell fate decision
Source: Cell Death Dis. 2017 Oct 19;8(10):e3130–. doi: 10.1038/cddis.2017.492 (PMC5682658; doi:10.1038/cddis.2017.492)
Supplement: Supplementary Material [file cddis2017492x1.docx]

**Supplementary Information**

**p53 dynamics orchestrates with binding affinity to target genes for cell fate decision**

Mengqiu Wu^1,2^ ^†^, Hui Ye^1,^ ^†^, Zhiyuan Tang^3,^ ^†^, Chang Shao^1^, Gaoyuan Lu^1^, Baoqiang Chen^1^, Yuyu Yang^1^, Guangji Wang^1,*^ and Haiping Hao^1,*^

^1^ Key Laboratory of Drug Metabolism and Pharmacokinetics, State Key Laboratory of Natural Medicines, China Pharmaceutical University, Tongjiaxiang #24, Nanjing, Jiangsu, 210009, China

^2^ Department of Nephrology, Children's Hospital of Nanjing Medical University, Nanjing, Jiangsu, 210008, China

^3^ Department of Respiratory Medicine, Affiliated Hospital of Nantong University, Nantong, Jiangsu, 226001, China

^*^ Correspondence: haipinghao@cpu.edu.cn (H.H), Tel: 86-25-83271179; guangjiwang@hotmail.com (G.W), Tel: 86-25-83271128

^†^These authors contributed equally to this work.

**SI includes:**

Figures S1-S5

Supplemental Figure Legends

Table S1

Supplemental experimental procedures

Movies S1-S3

**Figure S1**

**
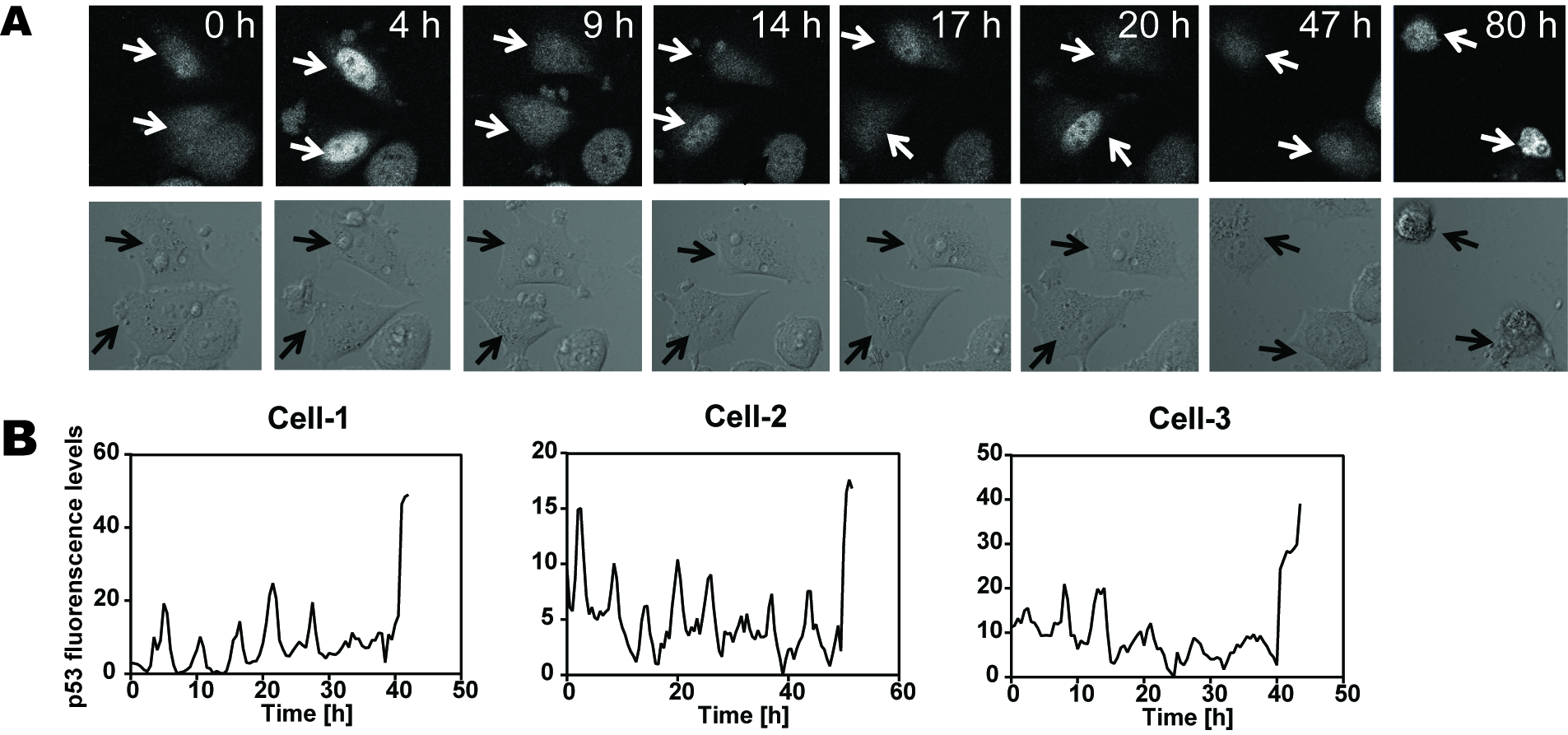
**

**Figure S2**

**
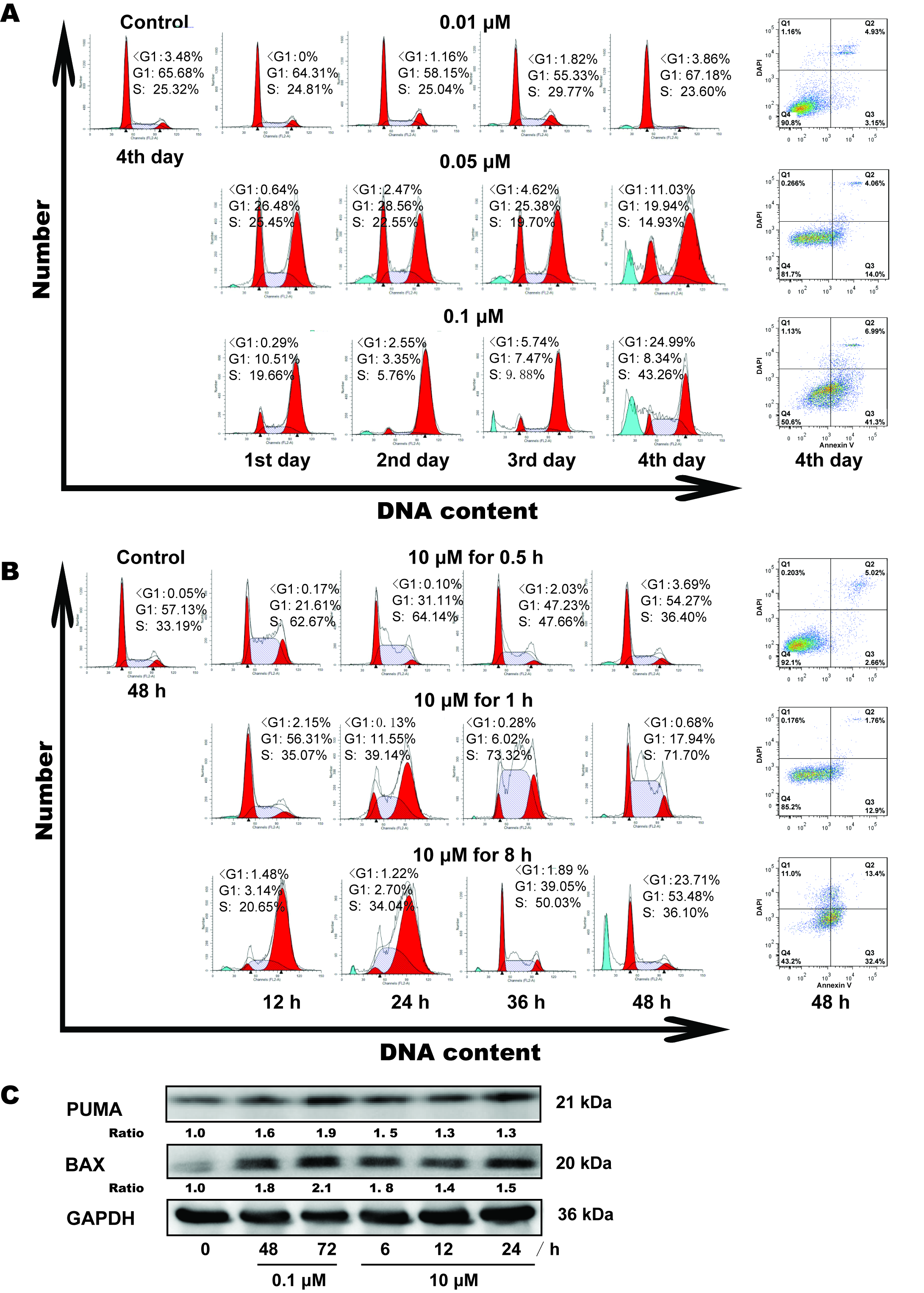
**

**Figure S3**

**
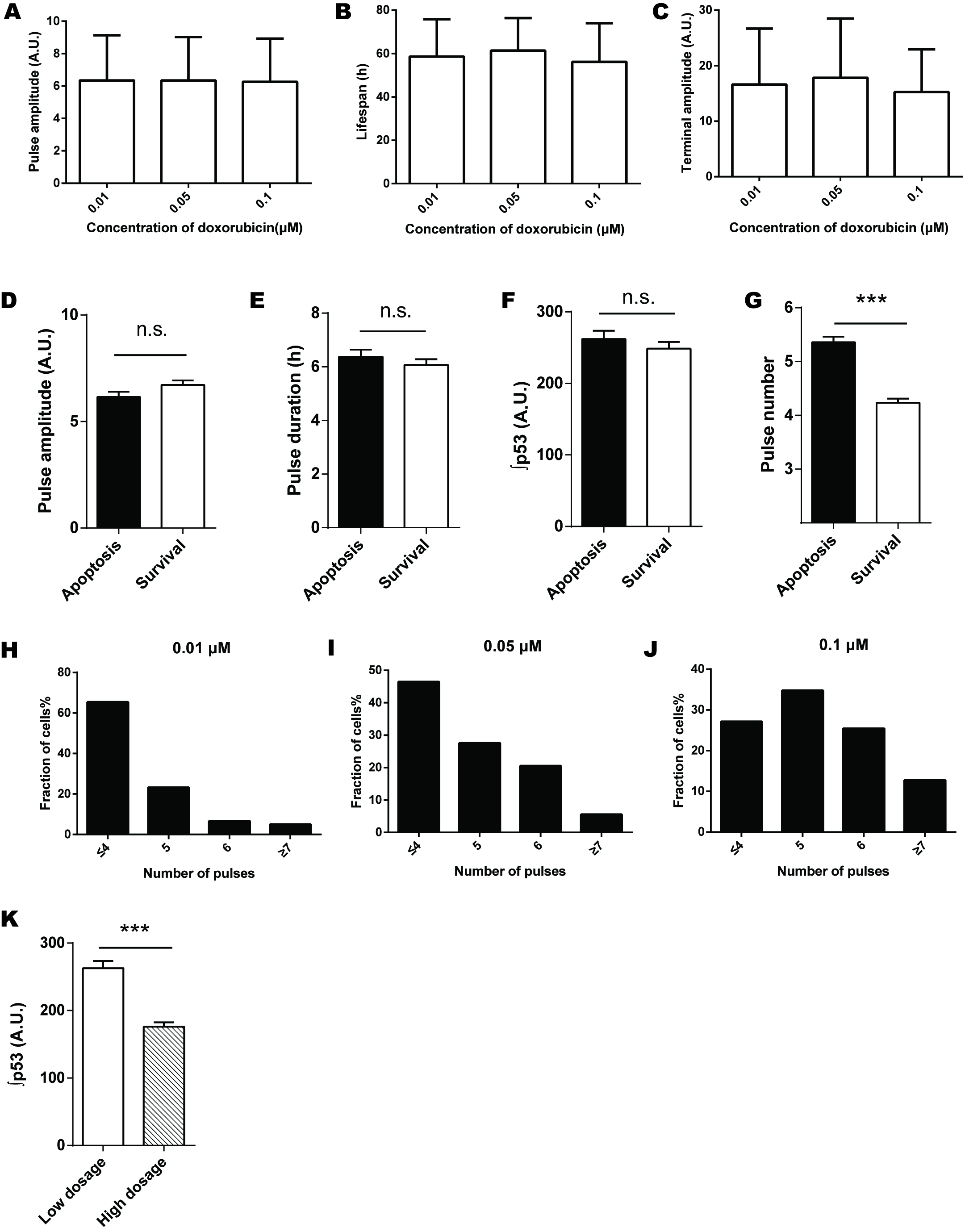
**

**Figure S4**

**
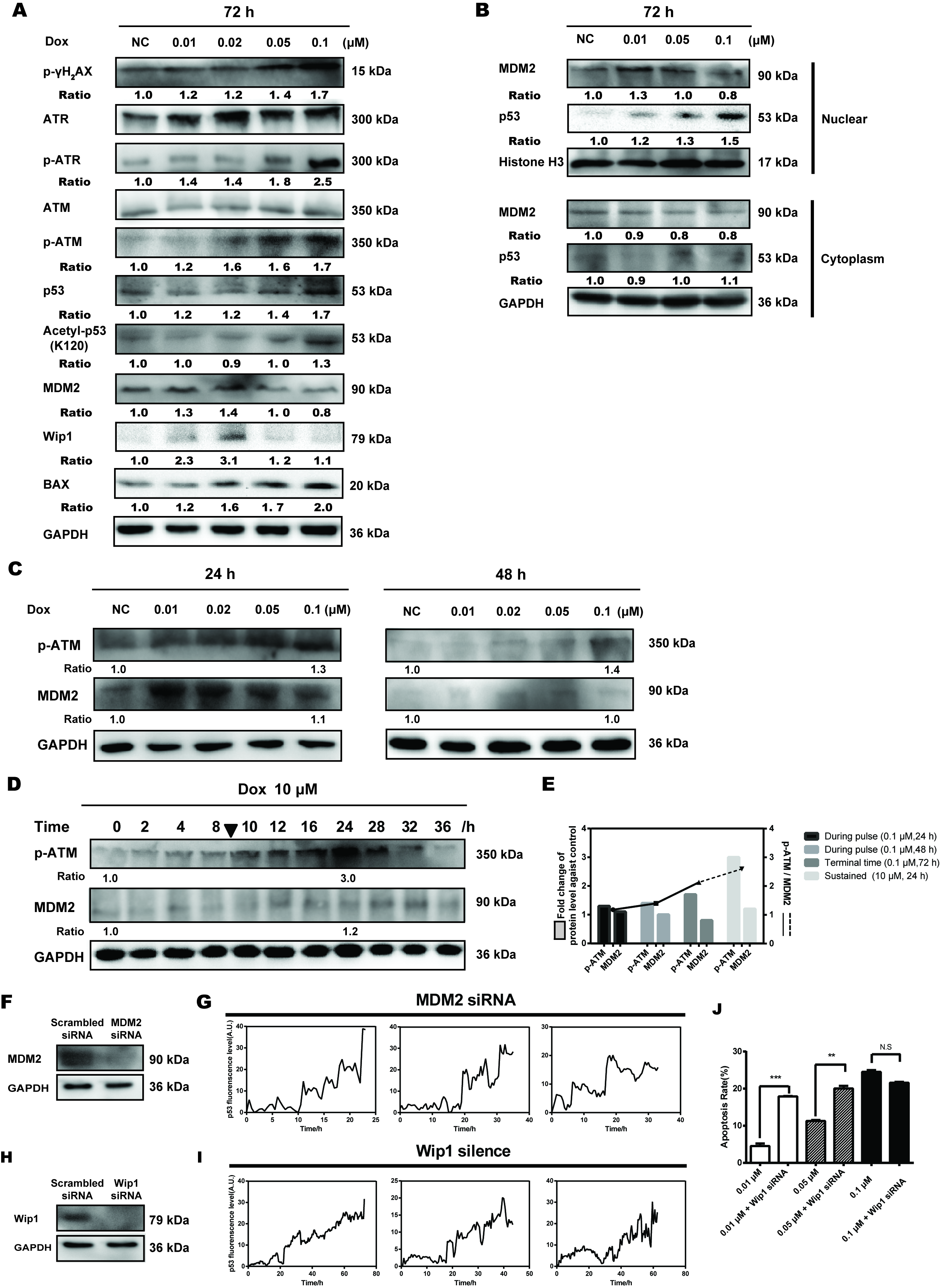
**

**Figure S5**

**
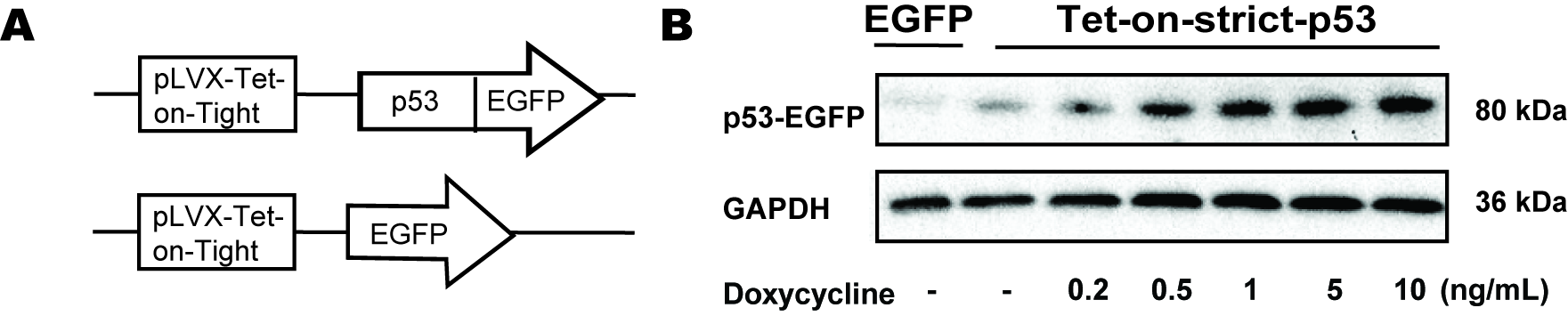
**

**SUPPLEMENTAL FIGURE LEGENDS**

**Figure S1. p53 dynamics induced by etoposide treatment**

Time-lapse images (A) and representative single-cell traces (B) of p53-Venus level in MCF7 cells following 1 μM etoposide treatment.

**Figure S2. Dynamic features and accumulation time combinatorially determine cell fates.**

**(A and B)** Dose and duration-dependency of mild (A) and acute (B) Dox-induced cellular apoptosis. DNA content of cells was measured using PI staining by flow cytometry at 1st~4th day after a mild Dox treatment and 12~48 hrs after an acute Dox treatment. Cellular apoptosis was also measured using Annexin V/DAPI staining at the ending time point. Control cells were cultured in drug-free medium (0.1% DMSO) and collected for analysis at indicated time. (C) Western blot analysis of BAX and PUMA after the mild and acute Dox-treatment at indicated time. Control cells were cultured in drug-free medium (0.1% DMSO) for 24 hrs. The immunoblotted intensities of each protein compared to GAPDH were quantified by densitometry and expressed in relative fold change to the control group.

**Figure S3. E∫p53, rather than pulse number or ∫p53, controls cell fate.**

**(A-C)** Pulse amplitude (A), lifespan (B) and terminal amplitude (C) held constant across cells that exhibited terminal pulse under the treatment of 0.01, 0.05 and 0.1 μM Dox within 96 hrs. Number of cells (n) = 121 (0.01 μM), n= 127 (0.05 μM) and n= 118 (0.1 μM).

**(D-G)** The average pulse amplitude (D), pulse duration (E), integrated p53 (∫p53) level (F) and pulse number (G) before terminal pulse in apoptotic (n= 227) and survival (n= 139) cells. Statistical analysis was performed using pooled data collected from cells under Dox treatments of 0.01, 0.05 and 0.1 μM. Data are represented as mean± SEM; ***p< 0.001.

**(H-J)** The fraction of cells showing ≤4, 5, 6 and ≥7 pulses following different treatments of Dox for 96 hrs in a concentration of 0.01 μM (H), 0.05 μM (I) and 0.1 μM (J). The cell fraction in each bin was calculated by summarizing the number of cells showing corresponding number of pulses followed by dividing them by the total number of cells included in the conducted experiments. The total number of cells= 366.

**(K)** ∫p53 level of apoptotic cells induced by low dosage of Dox (0.01, 0.05 and 0.1 μM) or high dosage of Dox (5, 10 and 20 μM). Number of cells were n= 139 (low dosage) and n= 214 (high dosage), respectively. Error bars represent SEM and unpaired student’s t test was conducted to determine the significance;***p< 0.001.

**Figure S4. Repression of p53 feed-back loops facilitates cell apoptosis by promoting the accumulation of p53.**

**(A)** Western blot analysis of phospho-γH2AX (a sensitive marker of DNA double-strand breaks), p53 upstream kinases (ATM and ATR), proteins involved in p53 feed-back loops (p53, MDM2, Wip1) and BAX (pro-apoptosis protein) after indicated Dox treatments for 72 hrs, when most cells show terminal pulse. The immunoblotted intensities of each protein compared to GAPDH were quantified by densitometry and expressed in relative fold change compared to normal control (NC).

**(B)** Nuclear translocation of p53 under indicated Dox treatments for 72 hrs. p53 and MDM2 levels in the nucleus and cytoplasm of MCF7 cells were determined by the corresponding immunoblotted bands, respectively. Histone H3 and GAPDH were used as loading controls for nuclear and cytoplasmic proteins, respectively. The immunoblotted intensities of p53 and MDM2 compared to loading controls were quantified by densitometry and expressed in relative fold change compared to NC.

**(C-E)** Relative expression levels of p-ATM, MDM2 and the ratio of p-ATM/MDM2 during p53 pulsing, terminal and monotonic pulse. The immunoblotted intensities of p-ATM and MDM2 compared to GAPDH were quantified by densitometry and expressed in relative fold change to NC. Triangle in (D) indicated the time when Dox was withdrawn.

**(F)** Expression levels of MDM2 following transfection with corresponding siRNA (13.3 nM) for 36 hrs. GAPDH were used as loading controls.

**(G)** Representative single-cell traces of MCF7-p53 Venus cells transfected with siRNA targeting MDM2 and 0.1 μM Dox treatment. Dox was added after 36 hrs of silencing.

**(H)** Expression levels of Wip1 following transfection with corresponding siRNA (13.3 nM) for 36 hrs. GAPDH were used as loading controls.

**(I)** Representative single-cell traces of MCF7-p53 Venus cells transfected with Wip1-targeted siRNA and followed by a 0.1 μM Dox treatment.

**(J)** The apoptotic rate of Dox-treated MCF7 cells was increased by the silencing of Wip1. Data are presented as mean± SD from three independent experiments. Unpaired student’s t-test was conducted to determine the significance; **p< 0.01, ***p< 0.001.

**Figure S5*.* A threshold exists for p53 to bind to and transactivate pro-apoptotic genes.**

**(A)** Schematic representation of the tet-on-strict-p53*EGFP and tet-on-strict-GFP (control) vector system.

**(B)** Immunoblot analysis of the p53-EGFP expression levels in MCF7 cells transfected with tet-on-strict-p53*EGFP or control vectors followed by induction with 0.2, 0.5, 1, 5 and 10 ng/mL doxycycline for 48 hrs. Lane 1 represents the parental MCF7 cells transfected with control plasmid without treatment of doxycycline, and Lane 2 exhibits cells transfected with tet-on-strict-p53*EGFP plasmid yet without treatment of doxycycline (Lane 2) for 48 hrs. GAPDH was used as a loading control.

**Table S1. Sequences of primers or probes used in quantitative reverse transcriptase PCR and single cell RNA FISH microscopy.**

| **Quantitative reverse transcriptase PCR** | | |
| --- | --- | --- |
| **Name** | **Forward primer(5’-3’)** | **Reverse primer(5’-3’)** |
| ***XPC*** | GCCAGTGAACAAGATAACC | GACCAATTCCTCATCATCT |
| ***p21*** | GTGGACCTGGAGACTCTC | TTCCTCTTGGAGAAGATCAG |
| ***BAX*** | TTGTCGCCCTTTTCTACT | CGGAGGAAGTCCAATGTC |
| ***APAF1*** | ATGAAGCCATGTCTATAAGTGTT | GCACCTTAACGTCCTTCTG |
| ***GAPDH*** | AACAGCCTCAAGATCATC | CACGATACCAAAGTTGTC |
| **Single cell RNA FISH microscopy** | | |
| ***p21* (conjugated to TET)**  ggggttatctctgtgttaggggtatatgatgggggagtagatctttctaggagggagacactggcccctcaaatcgtccagcgaccttcctcatccaccccatccctccccagttcattgcactttgattagcagcggaacaaggagtcagacattttaagatggtggcagtagaggctatggacagggcatgccacgtgggctcatatggggctgggagtagttgtctttcctggcact;  ***APAF1* (conjugated to AMCA)**  cttgatcttggatgatgtttgggactcttgggtgttgaaagcttttgacagtcagtgtcagattcttcttacaaccagagacaagagtgttacagattcagtaatgggtcctaaatatgtagtccctgtggagagttccttaggaaaggaaaaaggacttgaaattttatccctttttgttaatatgaagaaggcagatttgccagaacaagctcatagtattataaaagaatgtaaagg | | |

*XPC*: xeroderma pigmentosum, complementation group C; *p21(CDKN1A)*: cyclin dependent kinase inhibitor 1A; *BAX*: BCL2-associated X protein; *APAF1*: apoptotic peptidase activating factor 1. *GAPDH*: glyceraldehyde-3-phosphate dehydrogenase ; TET: Tetrachloro fluorescein; AMCA: 7-amino-4-methylcoumarin-3-acetic-acid.

**Movie S1.** Time-lapse series of cells demonstrating dual-phase p53 pulses. MCF7-p53 Venus cells were treated with 0.1 μM Dox. Movie duration: 88 hrs. Images were taken every 30 min.

**Movie S2.** Time-lapse series of cells demonstrating single-phase p53 pulses. MCF7-p53 Venus cells were treated with 0.01 μM Dox. Movie duration: 88 hrs. Images were taken every 30 min.

**Movie S3.** Time-lapse series of cells demonstrating sustained p53 accumulation. MCF7-p53 Venus cells were treated with 10 μM Dox for 8 hrs and cultured after removal of the drug. Movie duration: 28 hrs. Images were taken every 30 min.

**SUPPLEMENTAL EXPERIMENTAL PROCEDURES**

**Western Blot analysis**

Harvested cells were lysed with NP-40 lysis buffer in the presence of 1% protease inhibitors cocktail (Sigma, St.Louis, MO, USA). Total protein levels were quantified using the bicinchoninic acid (BCA) assay. Equal protein amounts were separated by SDS–PAGE using 6~12% gradient (Bis-Tris Midi Gel, Invitrogen) polyacrylamide gels and transferred onto PVDF membranes. Blots were blocked, followed by incubation with primary antibody and subsequently with secondary antibody coupled to peroxidase. The resulting immunoblotted bands were detected using a chemoluminiscence kit (Bio-Rad Laboratories, Hercules, CA) on a ChemiDoc XRS+ System (Bio-Rad). The used antibodies include: p53 (＃05-224, Millipore Corp., Billerica, MA, USA); Acetyl K120 p53 (＃ab78316) and MDM2 (＃ab16895) were purchased from Abcam (Cambridge, MA, USA); phosphorylated γH2AX (Ser139) (＃9718), ATM (＃2873), p-ATM (Ser1981) (＃4526), p-ATR (Ser428) (＃2853) and BAX (＃2772) were all purchased from Cell Signaling Technology (Danvers, MA, USA); PUMA (＃55120-1-AP, Proteintech Group, Chicago, IL, USA); Wip1 (＃sc-20712, Santa Cruz Biotechnology, Santa Cruz, CA, USA); ATR (＃A300-137A, Bethyl Laboratories, Montgomery, TX). GAPDH (＃5174, Cell Signaling Technology, Danvers, MA, USA) or Histone H3 (＃4499, Cell Signaling Technology, Danvers, MA, USA) were used as loading controls.

**Flow Cytometry Analysis of Cell Cycle and Apoptosis**

After treatment, cells in different cell cycle phases and apoptosis was determined by propidium iodide (PI) staining of DNA content. Data were acquired on a BD FACS Calibur (Becton and Dickinson, Franklin, NJ).

**Quantitative reverse transcriptase PCR**

Total RNA was extracted and reversely transcribed according to the manufacturer’s protocol (Applied Biosystems). Quantitative PCR was subsequently carried out using SYBR Select Master Mix (Applied Biosystems) on a Step One Plus PCR System (Applied Biosystems). The primers used for PCR amplification are listed in Table S1. Expression levels were normalized to GAPDH.

**RNAi**

MCF7-p53 Venus cells were transfected with scrambled siRNA and siRNA targeting MDM2 or Wip1 using RNAiMAX transfection Reagent (Invitrogen) according to the manufacturer’s instruction. siRNAs targeting Wip1 (sequences, UUGUGAGUGAGUCGAGGUCGUUUCC and GGAAACGACCUCGACUCACUCACAA) and MDM2 (sequences, UUACAGCACCAUCAGUAGGUACAGA and UCUGUACCUACUGAUGGUGCUGUAA) from Invitrogen were used at a final concentration of 13.3 nM.
